# Personal

**Published:** 1903-07

**Authors:** 


### PERSONAL.

Dr. G. A. Himmelsbach, of Buffalo, has removed his office and residence from 137 West Tupper Street to his new office and residence at 382 Elmwood Avenue. Telephones: Bell, Bryant 106; Frontier 687. Hours: 8 to 9 a.m.; 2 to 3 and 7 to 8 p.m.

---

Dr. Harvey R. Gaylord, of Buffalo, who has recently been ill with typhoid fever, has recovered therefrom, and sailed for Europe, June 20, 1903. Dr. Gaylord will occupy a position as complimentary assistant in the surgical clinic of Professor Mikulicz in Breslau, where he will spend at least a year. On his return to Buffalo, he will devote himself to the practice of surgery.

---

Dr. Montgomery A. Crockett, of Buffalo, has gone to Hamburg, Germany, where he is pursuing the study of general surgery.

---

Dr. G. H. A. Clowes, of the Gratwick research laboratory, Buffalo, is spending his vacation of three months in Europe.

---

Dr. Chauncey Pelton Smith, of Buffalo, has removed his office from 90 N. Pearl Street to 481 Franklin Street. Hours: 9 to 10 a.m., 2 to 3 and 8 p.m. Telephone, Tupper 412.

---

Dr. Frederick Preiss, of Buffalo, sailed for Europe, May 27, 1903, where he will spend some time in medical study in Great Britain and on the continent.

Dr. A. E. Sohmer, formerly of New York, has located at 1343 Jefferson Street, Buffalo. Hours: 8 to 9 a.m., 1 to 3 and 7 to 8 p.m. Telephone: Bell, Bryant 5016; Frontier, 3910.

---

Dr. Max Keiser, of Buffalo, is spending the summer in Europe. He has lately removed his office from 388 Franklin Street to 888 Main Street.

---

Dr. E. P. Hussey, of Buffalo, was elected vice-president of the American Institute of Homeopathy during its recent annual meeting at Boston.

---

Dr. A. L. Benedict, of Buffalo, has gone abroad for a six weeks' vacation.

---

Dr. Jefferson D. Griffith, of Kansas City, attended the Fourteenth International Medical Congress, recently held at Madrid, as one of the delegates from this country.

---

Dr. Richard Douglas, of Nashville, whose treatise on Surgical Diseases of the Abdomen has just issued from the press, was one of the delegates to the Fourteenth International Congress, held at Madrid in April, 1903.

---

Dr. L. H. Dunning, of Indianapolis, was elected chairman of the section on obstetrics and gynecology at the recent annual meeting of the American Medical Association.

---

Dr. Edwin Walker, of Evansville, Ind., has been appointed chairman of the business committee of the house of delegates, American Medical Association, for the current year.

---

Dr. John Herr Musser, of Philadelphia, president-elect of the American Medical Association, visited Buffalo, June 16, 1903, at which time he presented the subject of Cancer of the Lungs, before the District Branch of the State Medical Association. A banquet was tendered Dr. Musser in the evening at The Genesee, at which speeches were made by several representative physicians, including the guest of honor.

---

Dr. James W. Charters, of Buffalo, recently sailed by the Cunard Line for a tour in Europe.

Dr. T. D. Davis, of Pittsburg, was elected first vice-president of the American Academy of Medicine at its recent meeting at Washington.

---

Dr. Charles McIntire, of Easton, Pa., was again elected secretary of the American Academy of Medicine. At the last meeting he was president, and now goes back to his old place in which he has served so long and so capably.

---

Dr. Alonzo Garcelon, of Lewiston, Me., formerly governor of the Pine Tree State, may be named with great oppropriateness the Nestor of the American medical profession. Dr. Garcelon attended the recent meeting of the American Medical Association at New Orleans, at which, on May 6, his friends, in celebration of his ninetieth birthday, presented him with a loving cup and cane. The occasion was so rare that it served to add to the interest of the meeting.

---

Dr. F. Park Lewis, of Buffalo, has been appointed by Governor Odell, chairman of the commission, to investigate the condition of the adult blind in the State of New York.

---

Dr. Joseph W. Grosvenor was elected president of the Buffalo Academy of Medicine at the annual meeting held June 9, 1903.

